# In Situ Zymography Analysis of Matrix Metalloproteinases Activity Following Endodontic Irrigation Protocols and Correlation to Root Dentine Bond Strength

**DOI:** 10.3390/polym14173567

**Published:** 2022-08-30

**Authors:** Abayomi Omokeji Baruwa, Claudia Mazzitelli, Tatjana Maravic, Jorge N. R. Martins, Annalisa Mazzoni, António Ginjeira

**Affiliations:** 1 Department of Endodontics, Faculdade de Medicina Dentária, Universidade de Lisboa, Rua Professora Teresa Ambrósio, 1600-277 Lisboa, Portugal; 2 Department of Biomedical and Neuromotor Sciences, DIBINEM, University of Bologna, Via S. Vitale 59, 40125 Bologna, Italy

**Keywords:** zymography, chlorhexidine, matrix metalloproteinases, push-out bond strength, irrigating solutions, fiber post

## Abstract

The objective was to evaluate the effect of different root canal irrigating solutions on the activity of matrix metalloproteinases (MMPs), and correlation to the push-out bond strength (PBS) and nanoleakage expression (NL) in the root dentin. Seventy-two single-rooted teeth were treated endodontically and distributed into four groups (*n* = 6 for in-situ zymography, *n* = 10 for PBS, and *n* = 2 for NL per group) according to the irrigating solutions used: (I) saline (S); (II) 5.25% sodium hypochlorite (SH); (III) 5.25% SH + 10% citric acid (CA); and (IV) 5.25% SH + 10% CA + 0.2% chlorhexidine (CHX). After root canal obturation, post space was prepared to receive the glass fiber post. Dual-cure resin was used for luting and light polymerization was performed. The root/fiber post assemblies were sectioned and subjected to in situ zymography, and PBS and NL expression analysis tests. The enzymatic activity was quantified and expressed as a percentage of the green fluorescence, while fractographic evaluation was performed after PBS with a stereomicroscope, and data were statistically analyzed at *p* < 0.05. The zymography analysis shows high expression of MMPs in the middle third of the root in all groups, while the most abundant activity of MMPs following the irrigating solutions is observed in groups I and III, where saline and citric acid are used, respectively. Inversely, group IV, where chlorhexidine is the final rinse, records the lowest MMP activity with the highest PBS, and the statistical analysis of the groups are ranked as: IV > II > III > I (*p* < 0.05). The combination of SH, CA, and CHX results in lower expression of MMPs and higher push-out bond strength of fiber posts to root dentin, with no difference seen in the nanoleakage expression (*p* > 0.05); hence, this irrigation regime with chlorhexidine as a final rinse is more favorable than other combinations in ensuring optimal adhesion to root dentine.

## 1. Introduction

The objective of endodontic treatment is to satisfy biological requirements through the elimination of intra-canal microorganisms and their by-product toxins, and prevent further inflammation of peri-radicular tissues [1]. This goal is achieved through clinical protocols consisting of access cavity preparation, intra-canal cleaning and shaping, effective root canal irrigation, and three-dimensional canal obturation, followed by adequate restoration to prevent reinfection, and ensure dental structure robustness and strength to withstand the high occlusal forces [2,3]. Fiber posts are commonly used for that purpose in the case of extensive tooth structure loss [4]. The longevity of fiber post restorations is mostly related to the adhesion between the resin cement and radicular dentin [5]. Any impediment occurring between these two adhesive interfaces may contribute to a significant decrease in the longevity of the restorations [4,5,6,7,8,9,10].

The prognosis of an endodontic treatment and, in general, of the coronal reconstruction, is directly correlated to the irrigating solutions used between instrumentations. Indeed, they have the two-fold arduous role of detoxifying the root canal from microorganisms and necrotic tissues, and of removing the smear layer created during manual or rotary instrumentation [11]. These two goals are closely related to each other, since the persistence of the smear layer residues negatively affects the septic activity of the irrigants, eventually invalidating the seal of the root canal filling [12].

Endodontists have a wide range of choice of irrigation solutions and irrigation strategies, of which sodium hypochlorite (SH) represents the most-used and investigated root canal irrigant. Despite the well-recognized antimicrobial capabilities, it has been observed that SH only partially removes the smear layer, while it promotes the removal of dentinal proteins inside the root canal [12,13]. Moreover, when used in high concentrations or injected with high-pressure in the periapical tissue, SH can have toxic and irritative effects. Hence, in order to make the cleaning methods safer while retaining antimicrobial characteristics [14], alternative irrigants have been proposed over time, alone or in combination with SH, such as chelating agents (i.e., 17% ethylenediaminetetraacetic acid—EDTA), organic acids (i.e., 10% citric acid), and antibacterial compounds (i.e., chlorhexidine—CHX) [15].

These irrigating solutions play an essential role in the optimal chemo–mechanical preparation, by enhancing bacterial elimination and accelerating the removal of necrotic tissue and dentinal chips from the root canal [11]. In the past decades, multiple studies have reported and shown the effect of commonly used endodontic irrigating solutions on the radicular dentine, with the general consensus that while these solutions achieve the primary purpose of disinfecting the root canal space, their use has also led to some undesired effects on the dentine structure, such as decrease in dentin strength, microhardness, and changes in surface roughness [16,17]. Recently, an in vitro study also revealed that nano-hardness is adversely affected by irrigating solutions [18], and all of these have been linked to reduced bond strength.

Although there are several controversies and uncertainties in the literature as to the exact changes in the mechanical properties and mechanisms of these solutions that alter the dentine structure [18], preceding studies report that the concentration, volume, and pH of solutions adversely affect flexural strength [19], fractural resistance of roots [20], elasticity [21], and erosion of the dentine [22]. In addition, the changes in the structural components of the dentine caused by irrigating solutions also affect the adherence of microorganisms and the sealing ability of dental materials such as endodontic sealers and resin-based cement to the dentine [23,24], and this alteration in the interactions between treated dentine surfaces and sealing materials is due to the softening effect by the irrigants, which allows fast preparation and enables maneuverability in small, tight root canals [25].

Even though most of these studies were able to establish a cause-and-effect relationship between some of the widely available irrigating solutions used and the difficulty of bonding to the radicular dentine in a broader perspective, there is still no consensus regarding the preferred combination of irrigating solutions to be used during root canal treatment that could achieve the primary aim of debridement and similarly improve bonding. Nevertheless, the combination of sodium hypochlorite and a chelating agent such as EDTA or citric acid is generally the preferred combination for most clinicians [26]. However, for optimal adhesion to the root canal space, this combination alone may not be adequate, as extensive research in adhesion to coronal dentine reports the reactivation of the endogenous dentin matrix metalloproteinases (MMPs) following the use of acids such as in etch and rinse, self-etching adhesives, and demineralization procedures; these reactivated MMPs then act on the hybrid layer, causing the degradation of denuded collagen fibrils, thereby leading to bond failure, which negatively impacts the durability of the restoration [27,28,29].

Similarly in radicular dentine, the expression and activity of MMPs has been reported [30,31,32] with increased activity following the use of self-adhesives [30]. However, until recently, only one scientific report by Retana-Lobo et al. in 2021 [33] documented the effect of individual or combined irrigating solutions employed during endodontic treatment on the activity of MMPs in radicular dentine. Hence, the aim of the present study was to assess the effect of irrigating solutions used during root canal treatment on the activity of MMPs in the hybrid layer of bonded radicular dentine via in situ zymography and the correlation to the push-out bond strength and nanoleakage expression. The null hypotheses tested were that: (1) There is no difference in the activity of the MMPs following the use of different endodontic irrigating solutions; and (2) the irrigating solutions do not influence the push-out bond strength and the nanoleakage expression of fiber posts to radicular dentin.

## 2. Materials and Methods

The present laboratory study was approved by the Research Ethics Committee at the Faculdade de Medicina Dentaria, Universidade de Lisboa, Portugal (protocol number CEBD202101). Seventy-two intact single-rooted human permanent teeth with a single root canal (maxillary and mandibular), devoid of caries, cracks, or fractures, which were extracted due to orthodontic or periodontal reasons, with a minimum 13 mm of root length (measured between cement–enamel junction and the anatomic apex) were used for the purpose of the investigation. After extraction, the teeth were stored in artificial saliva prepared in the laboratory with pH 7.4 at 4 °C until use, for no more than 1 month.

### 2.1. Sample and Root Canal Preparation

The crown of each tooth was transversely cut at the cement–enamel junction (CEJ) using a low-speed handpiece with diamond cutting disc (KAVO Dental GmbH, Bismarckring, Germany) under constant water spray. All roots were examined visually and radiographically, to verify single canal anatomy and the absence of internal defects.

All root canal treatments were performed by a single operator (AOB) using a 6× magnification operative microscope (Leica, Wetzlar, Germany). The root canals were made patent with 15 K-file (Dentsply Sirona, Ballaigues, Switzerland) until they were visible at the apical foramina. The root canal space was mechanically shaped with rotary file Protaper Next X2 (Dentsply Sirona, Ballaigues, Switzerland), until a final apical diameter of 0.25 mm was achieved. The specimens were equally distributed into 4 groups (*n* = 18), according to the irrigating solutions used between instrumentation step: (I) 20 mL of saline solution (control group, SA) (Braun, Kronberg, Germany); (II) 20 mL of 5.25% sodium hypochlorite (SH) (Cerkamed, Stalowa Wola, Poland); (III) 20 mL of 5.25% SH + saline rinse + 2 mL of 10% citric acid for 1 min (CA) (Merck, Kenilworth, NJ, USA); and (IV) 20 mL of 5.25% SH, saline rinse + 2 mL of 10% CA for 1 min, saline rinse, and a final 2 mL of 0.2% chlorhexidine gluconate (CHX) (ISDIN, Barcelona, Spain).

All irrigations were performed with a 27-gauge side-vented needle (Henry Schein, New York, NY, USA) in a 5 mL syringe. Manual dynamic agitation with calibrated gutta percha corresponding to the apical diameter [34] was used with saline in group I and 5.25% SH in groups I–IV.

After irrigations, the roots were dried with paper points, and the canals were 3-dimensionally obturated using the continuous wave of condensation technique with AH Plus root canal sealer and gutta percha Protaper X2 cone 25/06 (Dentsply Sirona, Ballaigues, Switzerland). After obturation, the orifice of the canal was temporarily restored with cavit (3M, Seefeld, Germany), and the roots were wrapped in a gauze wet with artificial saliva and stored at 37 °C for 72 h before post space preparation.

After storage, the temporary obturation was removed and the coronal/middle gutta-percha was eliminated with heated endodontic plugger (EQ-V pack, Meta Biomed, Ochang, South Korea), leaving 4 mm of apical sealing. A 9 mm post space was prepared using disposable drills (Dentsply Sirona, Ballaigues, Switzerland) irrigated with distilled water and dried with paper cones. After checking the fit inside the post space, glass fiber tapered posts (X-post #2, tip diameter of 0.8 mm; Dentsply Sirona, Ballaigues, Switzerland) were cleaned with 90% alcohol and air-dried. The root canals in all groups were etched with 37% H_3_PO_4_ (Kerr, Orange, CA, USA) for 15 s, water-rinsed, and dried with absorbent paper points. Following the manufacturer’s instructions, an equal quantity of the primer and the catalyst of the etch-and-rinse adhesive Prime & Bond XP (Dentsply Sirona, Ballaigues, Switzerland) was mixed, and applied with disposable micro brushes, both in the root canal and on the fiber post surface for 5 s, and then air-dried. According to manufacturer’s instructions, the adhesive was left unpolymerized, on both the root canal and on the fiber post.

**Figure 1:**
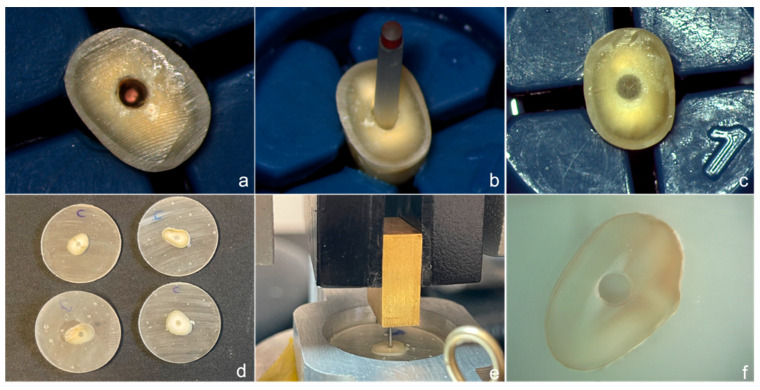
Images showing procedure: (**a**) root canal after obturation; (**b**) Glass fiber post try-in; (**c**) fiber post cementation; (**d**) One mm slice sections in acrylic blocks; (**e**) Mounted section prior to push-out testing; (**f**) and after testing.

A dual-cure resin cement Core X flow (Dentsply Sirona, Ballaigues, Switzerland) was used for fiber post cementation. The resin cement was injected by means of a disposable endodontic tip into the root canal. Then, the post was gradually inserted and held in position with a tweezer for 2 min. After fiber post insertion, the excess resin was removed with a micro brush and light polymerization was performed through the fiber post for 40 s with a mini LED (Satelec Acteon, Merignac, France) curing light (output: 1.250 mw/cm^2^) (Figure 1a–c). The specimens were stored in gauze immersed in wet artificial saliva at room temperature of 37.5 °C for at least 24 h.

### 2.2. Root Sectioning

After 24 h of laboratory storage, all specimens were embedded in a chemically cured acrylic resin (Henry Schein, New York, NY, USA) using cylindrical plastic molds. After complete setting of the resin, the blocks were sectioned using a diamond wheel mounted on a precision saw machine (Isomet 1000, Buehler Ltd., Bluff, IL, USA) under copious water cooling, to obtain 1 mm-thick slices (Figure 1d). Of these, the first coronal slice was discarded. The apical third of the root canal was filled with gutta-percha (the apical plug) and, therefore, not considered for the push-out bond strength test.

### 2.3. In Situ Zymography

The sections of the root (coronal, middle thirds) were identified and fixed to a microscope slide using cyanoacrylate glue. These sections were then flattened and polished by means of a grinding device (LS2; Remet, Bologna, Italy) under copious water irrigation, with a series of graded silicon carbide abrasive papers with different fineness (180-, 600-, 1200-, 2400-, and 4000-grit) to obtain an approximately 50 μm-thick section. Immediately after, the in situ zymography of the prepared samples was performed, following the protocols previously described by Mazzoni et al. 2012 [35].

On each root section glued to the glass slide, 50 µL of fluorescent gelatin mixture was applied, ensuring full immersion of the mixture prior to being covered with a coverslip, which was secured on the edge with a self-cure nail varnish. Thereafter, all slides were stored in a humid chamber at 37 °C, and protected from light.

The enzymatic gelatin activity of each sample on the microscope slide was assessed based on hydrolysis of the quenched fluorescein-conjugated gelatin as the MMP substrate (E-12055, Molecular Probes, Eugene, OR, USA), and evaluated under a multi-photon confocal laser scanning microscope (LSM 5 Pa: Carl Zeiss, Oberkochen, Germany) with excitation and emission wavelengths of 495 nm and 515 nm, respectively. To ensure optimal identification of the optimum incubation period, fluorescent images were obtained between 1 h and 3 days.

Multiple optical sections of 85 µm-thick were acquired from different focal planes from each root section, which were stacked, quantified, and processed using ZEN 2010 software (Carl Zeiss, Oberkochen, Germany) before analysis. The enzymatic activity differences from the experimental groups, represented by the fluorescence between the dentine and cement (within the HL), was quantified, and expressed as a percentage of the green fluorescence using ImageJ software (National Institutes of Health, Bethesda, MD, USA). To facilitate consistency in the measurement of all images for the groups analyzed, five measurements at different sites on each image was recorded using a standardized rectangular selection that was created and saved on the image software. For the analysis, 720 measurements were recorded from a total of 144 collated images for all groups.

### 2.4. Push-Out Bond Strength Test

Thirty-six slices in each group (18 coronal and 18 middle third sections) were included for the push-out test, which was performed using a universal testing machine (Instron 4502, MA, USA) at 0.5 mm/min crosshead speed, until fiber post debondings. The posts were pushed-out with adapted cylindrical plungers of different diameters (0.8, 1.0, and 1.5 mm), which were selected according to the size of the post and the coronal and middle thirds sections, and to ensure that the plunger contacted only the fiber post (Figure 1e). For this purpose, the coronal surfaces of the slices were marked to facilitate accurate placement of the specimen into the testing machine, and ensure that the force was applied from an apical–coronal direction. To determine the bond strength in megapascals (MPa), the load at failure recorded in N was divided by the area of the bonded interface, which was calculated using the equation: *a 1⁄4 2pr h*, where *p* is the constant 3.14, r is the post radius, and h is the thickness of the slice in mm.

**Figure 2:**
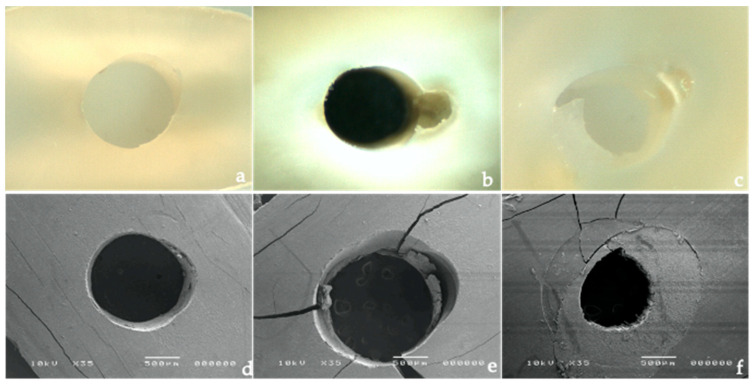
Failure modes of samples under the stereomicroscope ×10 magnification: (**a**) shows adhesive failure at the dentine (AD) by the clean wall; (**b**) adhesive failure at the resin/fiber post (AP) remnant of the resin cement in the canal walls; (**c**) mixed failure (M). Corresponding scanning electron microscopy images X35: (**d**) adhesive failure at the dentine (AD); (**e**) adhesive failure at the resin/fiber post; (**f**) mixed failure.

After testing, each specimen was examined under a stereomicroscope (Meiji Saitama, Japan) at 10× magnification (Figure 1f). The failure mode was classified as: A: adhesive between the resin cement and dentin (AD), or at the resin cement/fiber post level (AP); C: cohesive (within the cement); M: mixed, which is a combination of adhesive and cohesive failures occurring simultaneously (Figure 2a–c). Failures that occurred solely inside the post or dentin do not exceed the 3% of the total number of tested sections, and these were eliminated from the analysis.

### 2.5. Scanning Electron Microscopy Analysis

Two representative specimens per group were used for observational analysis under a scanning electron microscope (SEM). For this purpose, each specimen was fixed in a 2.5% glutaraldehyde 0.1 M cacodylate buffer for 3 h, and then dehydrated in ascending concentrations of ethanol (2 × 10 min of 50, 70, 80, 90, 95, and 100% ethanol). The ethanol was further slowly exchanged with HMDS, and the specimens were subsequently air-dried, gold–palladium-sputter-coated, and subjected to SEM analysis (JSM-5200, Jeol, Japan). The images obtained from the SEM evaluation are presented in Figure 2e–f.

### 2.6. Nanoleakage Expression

Evaluation of nanoleakage within the resin–dentin interface was performed as previously described by Tay et al. [36]. From each root section, 1 mm-thick slices were obtained from the coronal and middle regions, and immersed in 50 wt.% ammoniacal silver nitrite (AgNO_3_) solution for 24 h in the dark. Afterwards, the specimens were rinsed in distilled water, before immersion in the photo-developing solution for 8 h under fluorescent light, in order to reduce the silver ions into metallic silver grains within voids along the dentine-bonded interface.

Similar to the preparation of the in situ samples, each glued root section on a glass slide was flattened to obtain approximately 50 μm. The presence of the silver tracer was examined along the dentin-bonded interface using light microscopy (E800; Nikon, Tokyo, Japan), at 20× magnification. The interfacial nanoleakage expression was quantified based on the percentage of AgNO_3_ deposition at the adhesive surface, following the protocol described by Saboia et al. [37], using a scale 0–4 for evaluation: (0) no nanoleakage; (1) < 25%; (2) 25–50%; (3) 50–75%, and (4) > 75%.

### 2.7. Statistical Analysis

The statistical analysis was performed using the statistical package for the social sciences software 26 (SPSS Inc., Chicago, IL, USA) at a 0.05 level of significance. Due to non-homogeneity of the values recorded, the differences in MMP activity, push-out bond strength (Mpa) between different irrigating protocols, and the comparison between the root portion among the groups were tested using the Kruskal–Wallis test, and differences among nanoleakage scores were analyzed with the chi-squared test.

## 3. Results

### 3.1. In Situ Zymography

**Figure 3:**
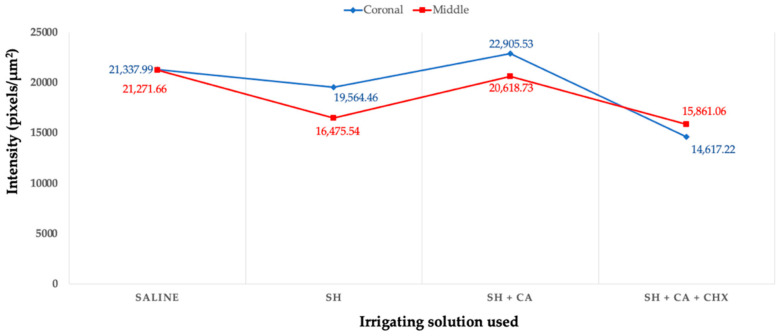
Line graph showing the values of gelatinolytic activity expressed as the intensity of green fluorescence in pixels/μm^2^ in the hybrid layers of the bonded radicular dentine of different irrigating solutions. [saline, sodium hypochlorite (SH), citric acid (CA), and chlorhexidine (CHX)].

The qualitative evaluation (Figure 3) of the enzymatic activity, as shown by the green fluorescence, shows differing intensity amongst the groups within the hybrid layer and dentinal tubules. The highest fluorescence intensity is seen in the control group (saline) and group III (SH + CA), while group IV, where chlorhexidine is used a final irrigating solution during the root canal treatment, shows the least intensity. Additionally, more enzymatic activity is seen in the coronal thirds than the middle for all groups, except in group IV which shows otherwise.

**Figure 4:**
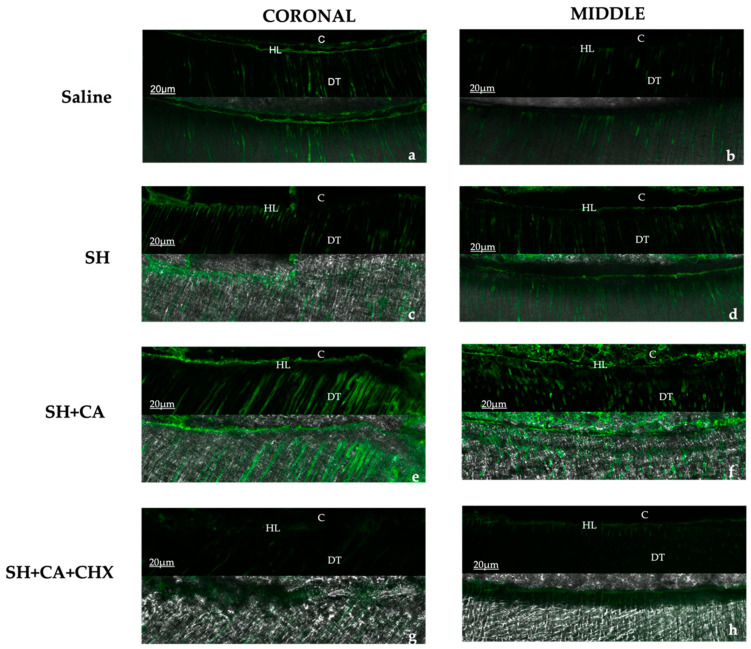
The sequence of representative in situ zymography images acquired with the multi-photon confocal microscope for the groups in the coronal and middle thirds, showing the green fluorescence as a result of the hydrolyzation of fluorescein-conjugated gelatin by the enzymes: saline (**a**,**b**); SH: sodium hypochlorite (**c**,**d**); SH + CA: citric acid (**e**,**f**); SH + CA + CHX: chlorhexidine (**g**,**h**). [HL: hybrid layer; DT: dentinal tubules; C: resin cement].

For the quantification of fluorescence intensity that is emitted by the hydrolyzation of fluorescein-conjugated gelatin, the measurement is limited to the zone between the dentinal wall and cement (hybrid layer), excluding the activities within the dentinal tubule. The quantification of enzymatic activity confirms the qualitative assessment, with the highest mean value recorded in group III > I > II > IV (Figure 4). There is a statistical significance (*p* < 0.05) amongst the irrigating solutions, but no significance when the root section and the type of irrigating solution used are compared between the groups.

### 3.2. Push Out Bond Strength Test

Table 1 shows the mean push-out bond strength values with standard deviation (SD) and failure mode classification among the groups in the coronal and middle root regions. The statistical analyses reveal that the irrigating solutions (*p* = 0.001), the root portion (*p* = 0.001), and the interaction between these two factors (*p* = 0.001) significantly influence the results (*p* < 0.005).

A common finding across all experimental groups is that the push-out bond strengths recorded in the middle regions are lower than those obtained in the coronal sector. However, differences are found between groups. Group IV (5.25% SH + 10% CA + 0.2 CHX) obtains the highest bond strength among the groups, both in the coronal and middle root portions (14.71 ± 1.82 MPa and 4.93 ± 0.38 MPa, respectively). Group I (only saline irrigation) records the lowest push-out values both in the coronal and middle root portions (5.19 ± 0.86 MPa, and 2.12 ± 0.38 MPa, respectively). When SH is used alone (group II), higher bond strengths are found than when SH is used in combination with CA (group III) in the coronal section (*p* < 0.05). Nonetheless, no statistically significant differences are observed between these two groups in the middle sector (*p* > 0.05).

The fractographic analysis reveals differences in failure mode among the groups. While group I (saline) registers a higher percentage of adhesive failures between the resin cement and dentin, group IV (SH + CA + CHX) results in a greater number of mixed fractures. Cohesive failures are also observed in all the experimental groups, with a slightly greater number in group II (SH).

### 3.3. Nanoleakage Expression

**Figure 5:**
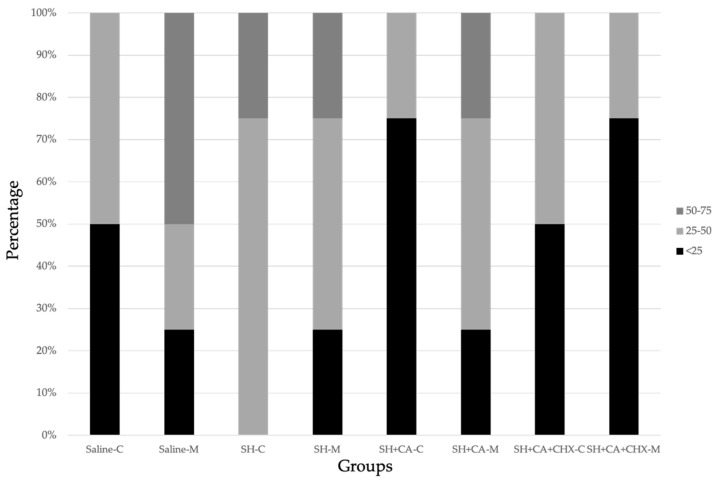
Percentage distribution of silver grains accumulation at the dentin–cement interface in the coronal (C) and middle (M) third sections of the tested groups. Saline (control), SH (5.25% sodium hypochlorite), SH + CA (10% citric acid), SH + CA + CHX (0.2% chlorhexidine).

**Figure 6:**
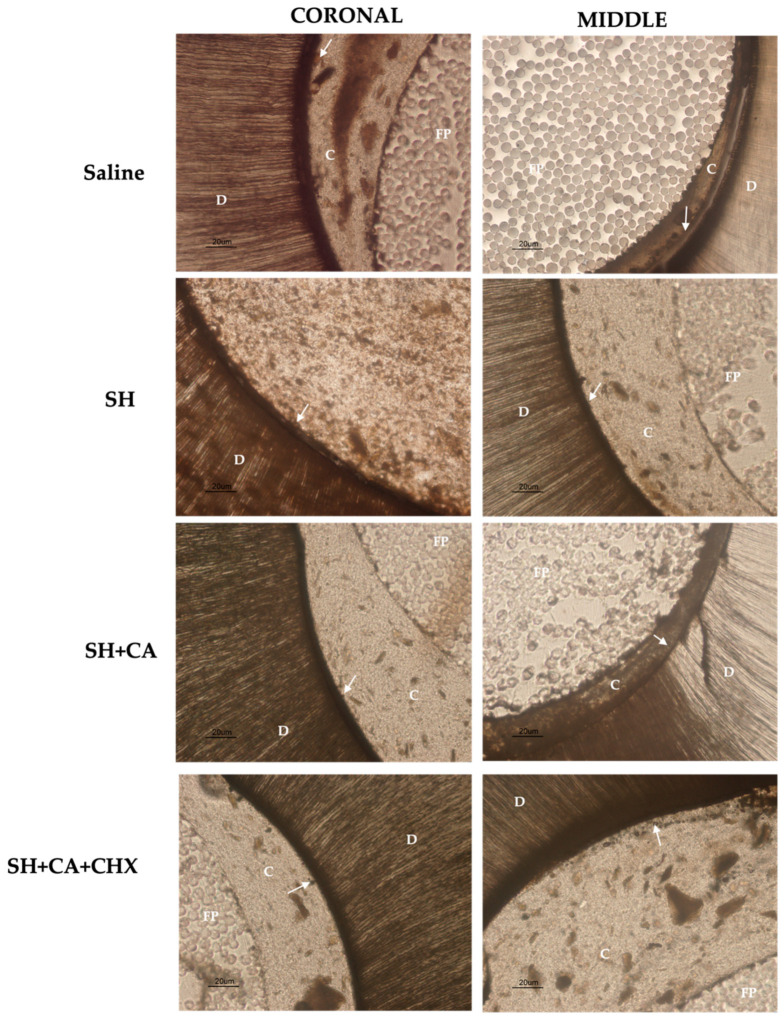
Representative nanoleakage expression images of all tested groups in the coronal and middle sections obtained under light microscope X20 showing the dentin (D), fiber post (FP), cement (C), and the deposits of silver nitrate at the adhesive surfaces (arrows).

Descriptive percentage distribution of the interfacial nanoleakage scores is presented in Figure 5 and Table 2. Representative light microscopy images are illustrated in Figure 6. No differences are observed in interfacial leakage scores among the groups, with a percentage of silver deposition ranging between 25–50%. Silver granule deposits lower than 25% are only observed in the coronal portion of group III (SH + CA), and in the middle portion of group IV (SH + CA + CHX).

## 4. Discussion

The decision of restoring a root-treated teeth with a post is to be considered when the remaining tooth structure is not adequate to support and retain the restoration, and, over the years, there have been reported successes in bonding fibre posts to root-treated teeth. However, there are still reports amongst clinicians regarding difficulties, although some of these challenges are associated with the type of adhesive and post used [38]. Particularly, reports show that the irrigating solutions used during endodontic treatment causes structural and chemical changes to radicular dentine, which, in turn, affects the bond strength to root dentine [18,33].

The present study shows that the activities of MMPs vary at different levels of the root, and the type of irrigating solutions used affected their expression, hence, the first null hypothesis is rejected. Although performed differently, the effect of the irrigating solution on MMPs was performed using an in situ zymography analysis of the hybrid layer after bonding, but our findings corroborate the results of Retana-Lobo et al. [33].

In the sections of the root, most enzymatic activity is seen in the middle sections of the root of all groups, which confirms the findings of Santos et al. [31], where stronger MMP-2 expression is recorded in the radicular dentine than coronal dentine. This could be associated with the structural changes in the morphology of radicular dentine in the apical direction, and the mineral concentrations and crystallite size of root dentine following the use of chemical agents during root canal treatment [9,39].

Similar to a previous study, we observe the highest enzymatic activities in the control groups where saline is used as the sole irrigating solution, and group III where sodium hypochlorite and citric acid are used in contrast to the other two groups. The possible explanation for the high activity seen in the control group could be due to the etch and rinse protocol and the adhesive used during the bonding procedure, as studies show that dentine demineralization and collagen exposure with the use of two-step-etch and rinse protocol and adhesive application significantly increases the activities of endogenous dentin MMPs [35].

The reduced activity seen in the group II with SH only confirms the reports that sodium hypochlorite causes the dissolution of organic dentine structures by infiltrating into the apatite encapsulated collagen matrix [19]. The effect of this destruction also alters the activity of the MMPs; although SH is also used in group III and the enzymatic activity is high, this can be attributed to the effect of the chelating agent (citric acid) used in this group, which causes additional demineralization and activation of latent endogenous MMPs. The effect of chelating agents, such as EDTA and citric acid, used in endodontic treatment protocol on dissolving the inorganic component of the smear layer is reported to also cause increased MMPs activities [33,40], although these chelating agents may initially have an inhibitory effect by binding to the zinc ions from the catalytic site of the MMPs with the subsequent removal of the calcium ions from the collagen matrices. This effect is reversible following their prolonged application and water solubility [40].

The influence of different root canal irrigation strategies on bond strength and nanoleakage expression of fiber posts was also tested to evaluate whether there are differences between the coronal and middle thirds of the root. The tested irrigating approaches record different bond strengths in the root in all groups. However, no significant differences in terms of nanoleakage expression are found between the groups. Consequently, the second null hypotheses must only be partially rejected.

The combination of SH + CA + CHX results in a higher push-out bond strength between the groups, both in the coronal and middle portions. This irrigating strategy was investigated in previous studies [41,42], but discordant results were reported, with this strategy not always resulting in favorable adhesion strength. Microscopy analyses reveal the formation of precipitation residues after endodontic irrigation with different chelating solutions, resulting in the occlusion of root dentin tubules [43]. Consequently, the obstruction of tubule orifices prevents the adequate penetration of the resinous monomers, possibly impairing adhesive strength and reducing the final seal of the restoration [43]. Therefore, in this study, rinses with saline solution after each irrigation solution (i.e., citric acid) or CHX were performed to prevent the precipitation of deleterious debris, and preserve clean and opened dentinal tubules. This procedural step could underlie the best adhesion results achieved by group IV (SH + CA + CHX). Furthermore, the saline rinse limits excessive dentin demineralization by solutions such as citric acid. Likewise, it limits any discolorations (brownish/reddish) that occur with the interaction of SH or CA with CHX.

The use of irrigation with CHX has gradually become more popular among endodontists, thanks to its substantivity, antibacterial capacity, and its ability as an MMPs inhibitor, which all enhance the bonding durability [44]. In support of several studies that CHX inhibits the activities of MMPs [45,46,47,48], and that, even when applied for 30 s, its inhibitory effect remains in the hybrid layer after 10 years [49], the findings in the current study show that the 0.2% CHX used as a final rinse solution following the combination of SH and citric acid records the least MMPs activity in the hybrid layer and the highest bond strength values.

According to the results obtained, entrusting the task of irrigating the endodontic space to the saline solution alone could be detrimental. Indeed, the control group records high MMPs activity and the lowest PBS values among the groups, both in the coronal and middle portion. The low MPa value in this group can be expected, because the saline has little or no tissue-dissolving capabilities, nor the ability to remove the smear layer that is created following the mechanical preparation of the root canal. Previous studies [50,51] show that tissue remnants with organic and inorganic debris in the smear layer affect adhesion to dentin. Despite this assumption, the use of adhesive systems that require a separate etching step with phosphoric acid gel (i.e., 37% H_3_PO_4_ as employed in this study) could favor the partial dissolution of debris not removed by irrigation with saline solution, and, thus, a better interaction with the resinous monomers, but cause the activation of endogenous MMPs leading to increased activities.

Even though the morphological evaluation of the root structure was not the subject of this study, alterations in the organic and inorganic components of the root dentin are reported after irrigations with SH, EDTA, and CA [52,53,54], resulting in higher interfacial microleakage and decreased bond strength between dentin and resin cements [55,56]. There are beneficial effects observed in our study, such as the fact that the combination of SH, CA, and CHX improves the push-out bond strength of fiber posts and inhibits the activities of MMPs when compared to other irrigation strategies.

In assessing and detecting the activities of MMPs in biological samples, several methods were developed, which includes substrate (gelatin) zymography, enzyme-linked immunosorbent assay (ELISA), and fluorescent resonance energy transfer-based measurement (FRET). Of all these techniques, substrate zymography is reported to be the most suitable and widely used method, due to its specificity in identifying and differentiating the latent and activated MMPs [57], although in situ zymography, which is a form of substrate (gelatin) zymography, is used in the current study, as this is currently the only viable means of assessing the activity of endogenous MMPs with the possibility of correlating it to the bond strength of root dentine. However, this method is reported to have low sensitivity [58], but its low cost, less time needed to complete, and practicality of digitally quantifying the fluorescence intensity with the aid of imaging software is advantageous over other protocols.

An unavoidable limitation in the current study is that the results from the three main methodologies could not be statistically combined, but it is possible to interpret and logically correlate the findings. Future studies are warranted to provide a complete perspective of the effects of the combination of irrigant solutions on the morphological and biochemical characteristics of root dentin.

## 5. Conclusions

Within the limits of this laboratory study, alternating irrigation with sodium hypochlorite, citric acid, and a final rinse with chlorhexidine inhibits the activity of endogenous MMPs and, in turn, yields superior bond strength when compared to only saline or sodium hypochlorite, whereas no differences in interfacial nanoleakage are recorded, in either the coronal or middle root portion.

## Figures and Tables

**Table 1:** Mean (MPa) push-out bond strength values (standard deviations) of each irrigating solution and failure mode distribution (%).

Groups	Mean (SD)		Failure Mode (%) (AD/AP/C/M)
	Coronal	Middle	
I (saline)	5.19 (1.52)	2.12 (0.67)	40/20/10/30
II (SH)	8.64 (3.18)	3.16 (0.49)	20/10/20/50
III (SH + CA)	6.78 (3.46)	3.43 (0.82)	30/20/10/40
IV (SH + CA + CHX)	14.71 (3.22)	4.93 (0.67)	10/10/10/70

Adhesive failure at the dentin side (AD) and at the fiber post interface (AP); C: cohesive failure within the resin cement; M: mixed failure.

**Table 2:** Percentage distribution of recorded nanoleakage values in the tested groups.

	Saline		SH		SH + CA		SH + CA + CHX	
	Coronal	Middle	Coronal	Middle	Coronal	Middle	Coronal	Middle
>75	0	0	0	0	0	0	0	0
50–75	0	50	25	25	0	25	0	0
25–50	50	25	75	50	25	50	50	25
<25	50	25	0	25	75	25	50	75
0	0	0	0	0	0	0	0	0

Saline, control; SH, sodium hypochlorite; CA, citric acid; CHX, chlorhexidine.

## Data Availability

The data from this study are available on request from the corresponding author.
